# Canine soft tissue sarcomas: the expression of RUNX2 and karyopherin alpha-2 in extraskeletal (soft tissues) and skeletal osteosarcomas

**DOI:** 10.3389/fvets.2024.1292852

**Published:** 2024-02-01

**Authors:** Leonardo Leonardi, Elisabetta Manuali, Antonello Bufalari, Ilaria Porcellato

**Affiliations:** ^1^Department of Veterinary Medicine, Università degli Studi di Perugia, Perugia, Italy; ^2^Laboratory of Comparative Veterinary Histopathology, Istituto Zooprofilattico dell'Umbria e delle Marche (IZSUM) “Togo Rosati”, Perugia, Italy

**Keywords:** canine, soft tissue sarcoma, extraskeletal osteosarcoma, skeletal osteosarcoma, immunohistochemistry, diagnostic tools

## Abstract

Extraskeletal osteosarcoma (EOS) is a malignant tumor producing bone matrix and/or chondroid material, without direct attachment to bone or periosteum. In humans and dogs, EOS is highly infiltrating, rapidly growing, often characterized by osteoid deposition and variable ossification, similar to primary skeletal osteosarcoma (SOS). In dogs, EOS arises from visceral and soft tissue locations, occasionally in trauma or foreign body sites, or in granulomas. Few data are currently available on the phenotype of these tumors. The present study aims to assess the expression RUNX2 and Karyopherin alpha-2 in EOS, comparing it with SOS and the data available from the human counterpart. Seventeen cases of canine osteosarcoma (13 EOS and 4 SOS) were retrospectively selected and submitted to immunohistochemistry for RUNX2 and Karyopherin alpha-2. Our results showed that, in EOS, RUNX2 is expressed in a mean of 73.07 ± 5.36 neoplastic cell nuclei, in face of a mean 36.15 ± 6.25 of Karyopherin alpha-2 positive nuclei. Osteoclasts, when present, were negative for both markers. No correlation was observed among the two markers (*p* > 0.05), nor statistically significant difference in quantitative expression was assessed comparing EOS and SOS groups. RUNX2 is expressed in canine EOS similarly to SOS and could be used as a diagnostic marker in a larger panel. Karyopherin alpha-2 is expressed in canine EOS and SOS similarly to human SOS and could be validated in future studies as an additional diagnostic marker. Further studies should be planned to evaluate the expression of these proteins as prognostic predictive parameters.

## Introduction

Extraskeletal Osteosarcoma (EOS) is a primitive osseous sarcoma defined by the production of osteoid or immature bone but arising in tissues other than bone without skeletal involvement (1–3). EOSs have been described in a variety of animal species and several tissues and organs such as the mammary gland first for frequency, followed by the spleen, skin and subcutis, intestine, muscle, liver (4), thyroid gland, urinary system, and salivary glands (1, 3). Like osteosarcomas of skeletal origin, extraskeletal osteosarcomas generally present as rapidly growing infiltrative masses, with areas of ossification at different stages of maturation, often also mixed with necrosis and hemorrhagic necrosis (1). Histologically, EOSs resemble skeletal osteosarcomas, with a frequent local recurrence and a high metastatic potential. EOSs have been diagnosed according to WHO guidelines and based on the primary location of the tumors and the certainty that the tumor is primitive from soft tissue and not from skeletal tissue. In dogs EOSs represent a highly malignant tumor with only <1 month median survival time after the first diagnosis (1, 5). A definitive histological diagnosis of osteosarcoma may not be straightforward, due to the high variability in cellular density of these tumors. Moreover, the highly unpredictable cellular morphology and the different histotypes of osteosarcoma can be challenging for the pathologist, particularly when chondroid matrix is present and chondrosarcoma must be included as a differential diagnosis.. In any case, EOS is always a tumor with high malignancy potential and frequent metastasis. While numerous biomolecular data present in the scientific literature describe various forms of primary skeletal osteosarcoma in many animal species, little has yet been done to better understand the most intimate and characterizing cellular aspects of animal EOS. During the last decade, numerous studies focusing on new markers associated with osteosarcoma pathogenetic mechanisms have emerged in human medicine, whereas in veterinary medicine, studies are still few (6). Among these factors, an emerging role has been attributed in some tumors to the expression of Karyopherin alpha2 (KPNA2), one of the seven members of the alpha karyopherin family. Its altered expression has already been described in various forms of cancer, but there are no specific and detailed data regarding its role in primary skeletal and extraskeletal osteosarcomas in dogs (7, 8). The same is true for RUNX2, an important transcription factor for skeletal development that is significantly involved in the activity of various bone cell components such as osteoblasts, multipotent mesenchymal cells, and chondrocytes (9). RUNX2 appears to be involved in many osteogenic and chondrogenic processes through the modulation of transcriptional activation and multiple signaling pathways (10). However, RUNX2 has also been identified as a potential co-factor in the biomolecular and genetic mechanisms that characterize tumorigenesis and neoplastic progression (11), through the modulation of mechanisms related to angiogenesis, metastasis, proliferation, cancer stemness and drug resistance (9, 10, 12). The purpose of this original research work is to try to identify new biomolecular and immunohistochemical aspects of canine extraskeletal osteosarcoma, focusing in this case on the expression of the two main markers indicated, never considered in veterinary medicine for these rare forms of spontaneous soft tissue tumors.

## Materials and methods

### Case selection

A total of 17 cases of canine osteosarcoma (13 extraskeletal and four skeletal) were retrospectively selected from the archives of the Department of Veterinary Medicine of the University of Perugia and the Istituto Zooprofilattico dell'Umbria e delle Marche (IZSUM) “Togo Rosati” – Laboratory of Comparative Veterinary Histopathology.

Cases were included in this study according to the following inclusion criteria.

- Histological diagnosis of skeletal osteosarcoma (SOS) or extraskeletal osteosarcoma (EOS).
- Available neoplastic tissue with an area >0.5 cm^2^.
- Primary tumors. For EOS, the presence of primary skeletal tumors had to be ruled out by X-rays or CT.

Cases were excluded if they had fixation artifacts or if they were decalcified for diagnostic purposes. The four SOS were randomly selected as a control group among the cases meeting the previously reported inclusion criteria.

### Antibody selection

The antibodies selected for the comparative study were RUNX2 (Santa Cruz, clone F-2) (13), and karyopherin alpha-2 (7) (Santa Cruz, clone B-9).

Considering that canine-specific antibodies against RUNX2 and karyopherin alpha-2 are not commercially available, we selected antibodies for humans, mice, or rats. The selection was based on the results of an *in-silico* analysis. We performed an alignment of the reported amino acid sequences, taking into account the residues used as targets to design the antibodies (www.uniprot.org) and thus selected and tested the antibody. Control tissues were canine osteosarcoma for RUNX2 and a canine testis for karyopherin alpha-2.

### Immunohistochemistry

In total, 5-μm sections from each FFPE block were cut and mounted on polarized slides, which were then dewaxed and dehydrated. Immunohistochemistry was performed on serial sections with antibodies against RUNX2 (heat-induced antigen retrieval in TRIS-buffer, pH 9.0; dilution 1:200) and karyopherin alpha-2 (heat-induced antigen retrieval in TRIS-buffer, pH 9.0; dilution 1:150). Immunohistochemistry was performed following the standard protocols previously reported (6). Negative controls were performed by omitting the primary antibodies and incubating the slides with PBS. RUNX2 was also useful to confirm the histological diagnosis (13). An immunohistochemical evaluation was performed blindly by one pathologist (IP) who evaluated the proportion of positive nuclei within the neoplastic population and assigned a percentage value (0–100%).

### Statistical analysis

Normality was assessed with a Shapiro–Wilk test for all continuous variables. Descriptive statistics were used to describe the data. Non-parametric tests were used to test hypotheses. The Mann–Whitney *U*-test was performed to assess differences between groups. Correlation analysis was performed using Spearman's test (ρ). Descriptive statistics were performed using Microsoft Excel; other statistical tests were performed using IBM SPSS (version 21).

## Results

### Selected cases

Of the 13 cases of EOS selected for this study, 10 were represented by female dogs (10/13; 76.9%), while only two dogs were male (15.4%). In one case the sex of the dog was not reported in our database and was therefore unknown. The mean age at the time of the histological diagnosis was 11.45 ± 2.3 years. In total, seven cases were primary EOS from the mammary gland (53.8%), all from female dogs, while four EOS were intrabdominal, with different origins (hepatic, splenic, vesical and one with an intrabdominal, non-organ-related origin). At the time of the histological diagnosis, the tumors had a mean major diameter of 8.55 ± 6.34 cm. Anamnestic data and the maximum diameter of the tumors selected for this preliminary study are reported in Table 1.

**Table 1:** Cases included in the study.

**Breed**	**Age**	**Sex**	**Location**	**Main diameter (cm)**	**Histological diagnosis**
Mixed breed	12	F	Intrabdominal	n/a	EOS
Mixed breed	n/a	n/a	Spleen	9,5	EOS
Mixed breed	13	F	Mammary gland	6	EOS
Deutsch Kurzhaar	11	F	Mammary gland	8	EOS
n/a	14	F	Mammary gland	4.5	EOS
Mixed breed	10	F	Mammary gland	6	EOS
Labrador Retriever	10	F	Liver	n/a	EOS
Mixed breed	15	F	Subcutis	n/a	EOS
Mixed breed	n/a	M	Subcutis	5	EOS
Miniature Schnauzer	10	F	Mammary gland	4	EOS
n/a	9	M	Urinary bladder	2.5	EOS
Mixed breed	14	F	Mammary gland	20	EOS
Mixed breed	8	F	Mammary gland	20	EOS
Mixed breed	9	M	Radiocarpal joint	5.5	SOS
Malinois	12	M	Mandible	4	SOS
Rottweiler	8	F	Tarsus	8	SOS
Mixed breed	7	F	Radiocarpal joint	3	SOS

### Immunohistochemistry

**Figure 1:**
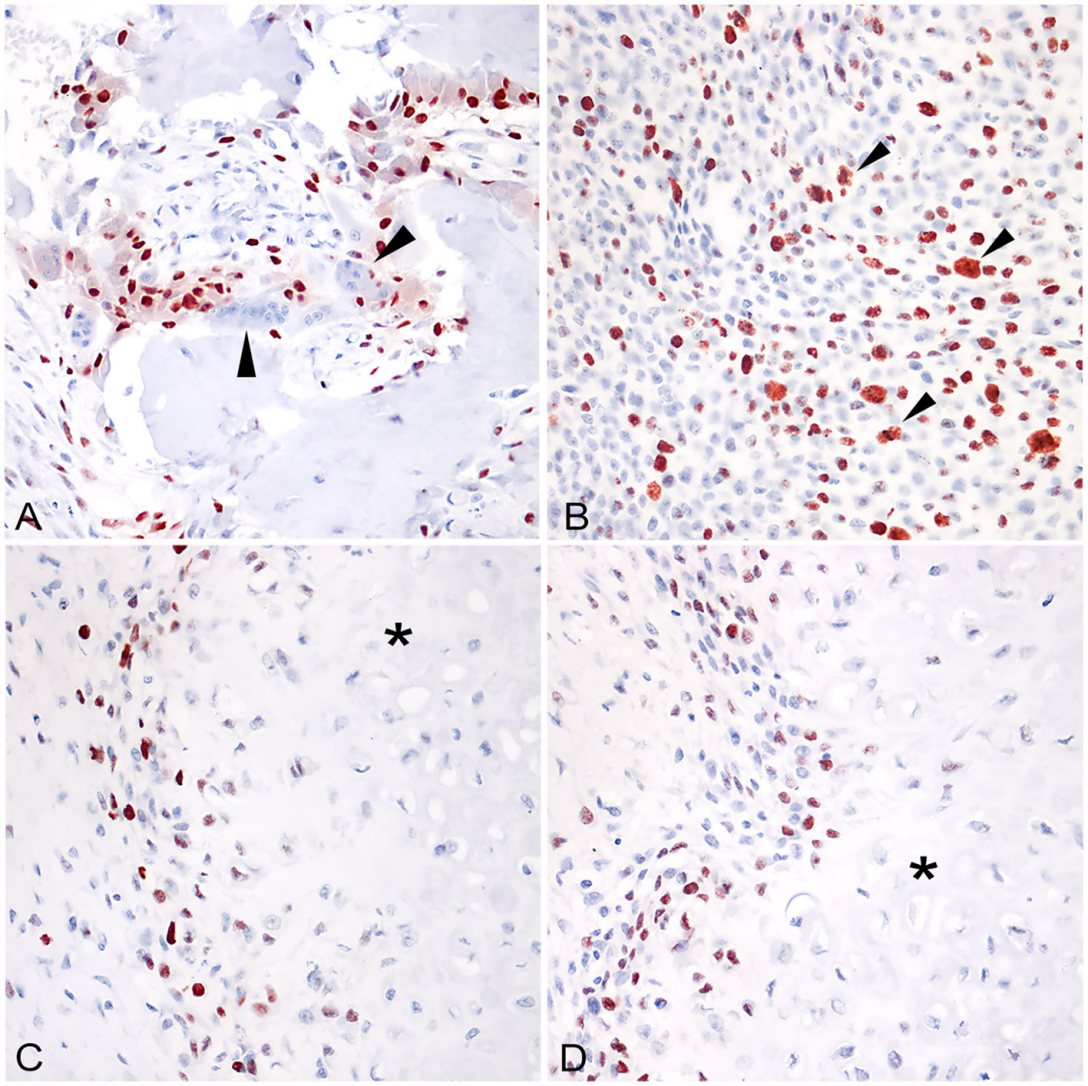
**(A)** Extraskeletal osteoblastic osteosarcoma (mammary gland), with abundant osteoid production. Osteoclasts were invariably negative for RUNX2 (arrowheads; 400x, HE); **(B)** Skeletal fibroblastic osteosarcoma (intrabdominal); the nuclear expression of karyopherin alpha2 is usually maintained in neoplastic cells during mitosis (arrowheads; 400x; AEC and hematoxylin). **(C)** Extraskeletal chondroblastic osteosarcoma (mammary gland), RUNX2 was not expressed in cells embedded in areas of chondroblastic differentiation (asterisk). **(D)** Extraskeletal chondroblastic osteosarcoma (mammary gland), karyopherin alpha 2 was not expressed in cells embedded in areas of chondroblastic differentiation (asterisk).

**Figure 2:**
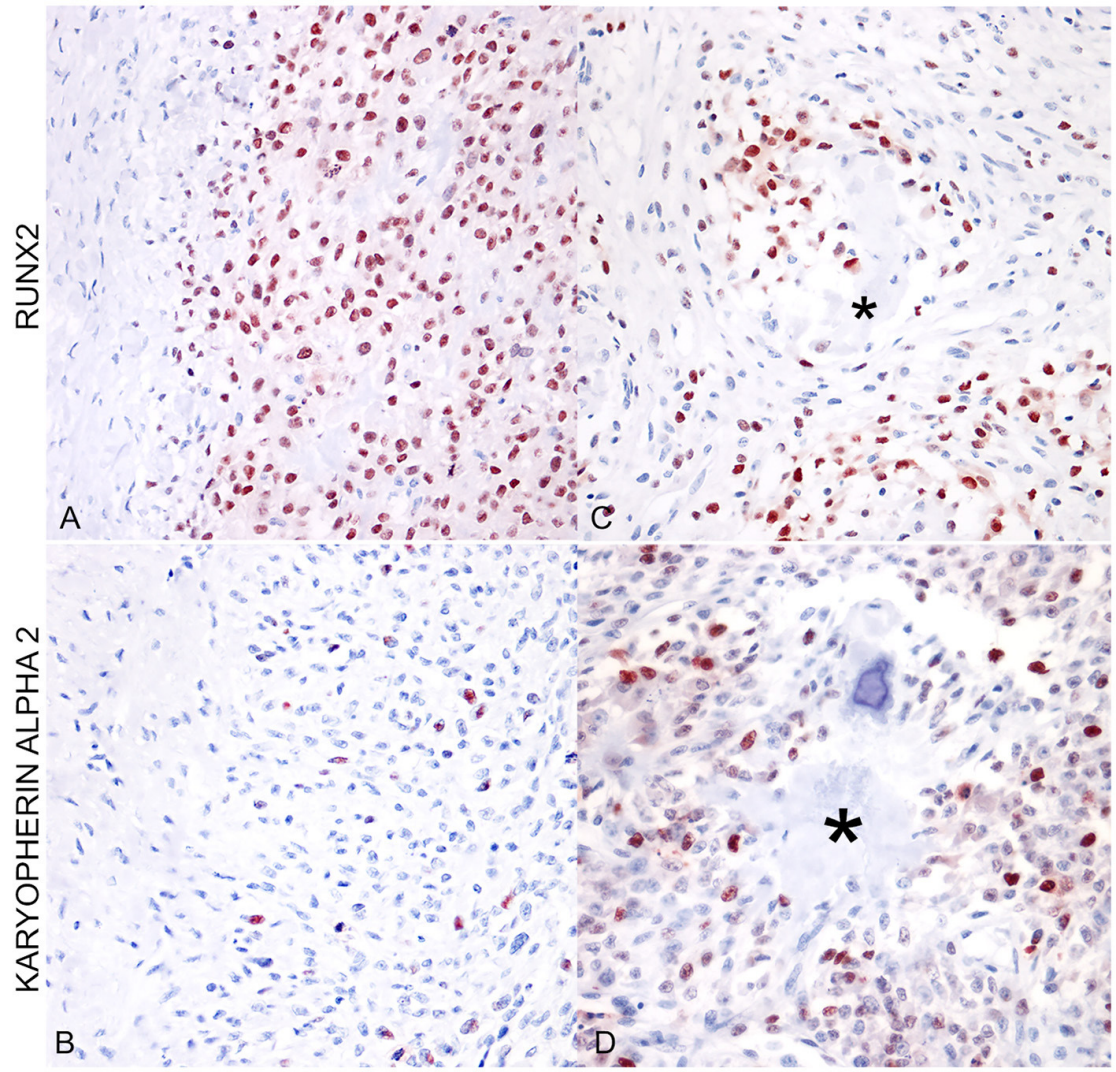
**(A)** Extraskeletal osteoblastic osteosarcoma (mammary gland). Immunostaining for RUNX2 shows a high percentage of positive nuclei (400x; AEC and hematoxylin). **(B)** Extraskeletal osteoblastic osteosarcoma (mammary gland). The same area as in **(A)** shows a lower percentage of positive nuclei for karyopherin alpha 2. **(C)** Extraskeletal moderately productive osteoblastic osteosarcoma (mammary gland). RUNX2-positive cells were more frequently localized around areas of osteoid deposition (asterisk). **(D)** Extraskeletal moderately productive osteoblastic osteosarcoma (mammary gland). The distribution of karyopherin alpha 2 is irregular and scattered among neoplastic cells, not associated with osteoid deposition (asterisk).

RUNX2 expression was observed as strong immunolabeling of the nucleus of neoplastic cells. The percentage of positive nuclei was variable with a mean of 73.07 ± 5.36. When applying to EOS the scoring suggested for SOS by Barger et al. was 1, 2/13 (15.38%) scored 2, 3/13 (23.1%) scored 3, while the remaining eight cases (61.53%) scored 4. Similarly, the expression of karyopherin alpha-2 was expressed in the nuclei of neoplastic osteoblasts of EOS and tested SOS and was also often observed in neoplastic cells during mitosis (Figure 1B). The percentage of positive nuclei was lower in EOS, when compared to RUNX2 (36.15 ± 6.25), but the intensity of the immunolabeling was always strong. Multinucleated giant cells occasionally present among neoplastic osteoblastic cells, and interpreted as osteoclasts, were always negative for RUNX2 (Figure 1A) and karyopherin alpha 2. Likewise, neoplastic cells embedded in areas of chondroblastic differentiation in chondroblastic EOS were negative for both markers (Figures 1C, D). Overall, RUNX2 showed a higher percentage of positive neoplastic cell expression when compared to karyopherin alpha 2 and the distribution of karyopherin alpha 2 positive cells was scattered among the other neoplastic cells (Figures 2A, B). RUNX2 expression was frequently observed in osteoblasts around areas of osteoid deposition, in contrast to karyopherin alpha 2 (Figures 2C, D), in both EOS and SOS. No correlation was observed between the expression of the two markers in the analyzed samples (*p* > 0.05), nor was there a statistically significant difference in terms of the quantitative expression of the two markers assessed between the EOS and SOS groups.

## Discussion and conclusions

**Figure 3:**
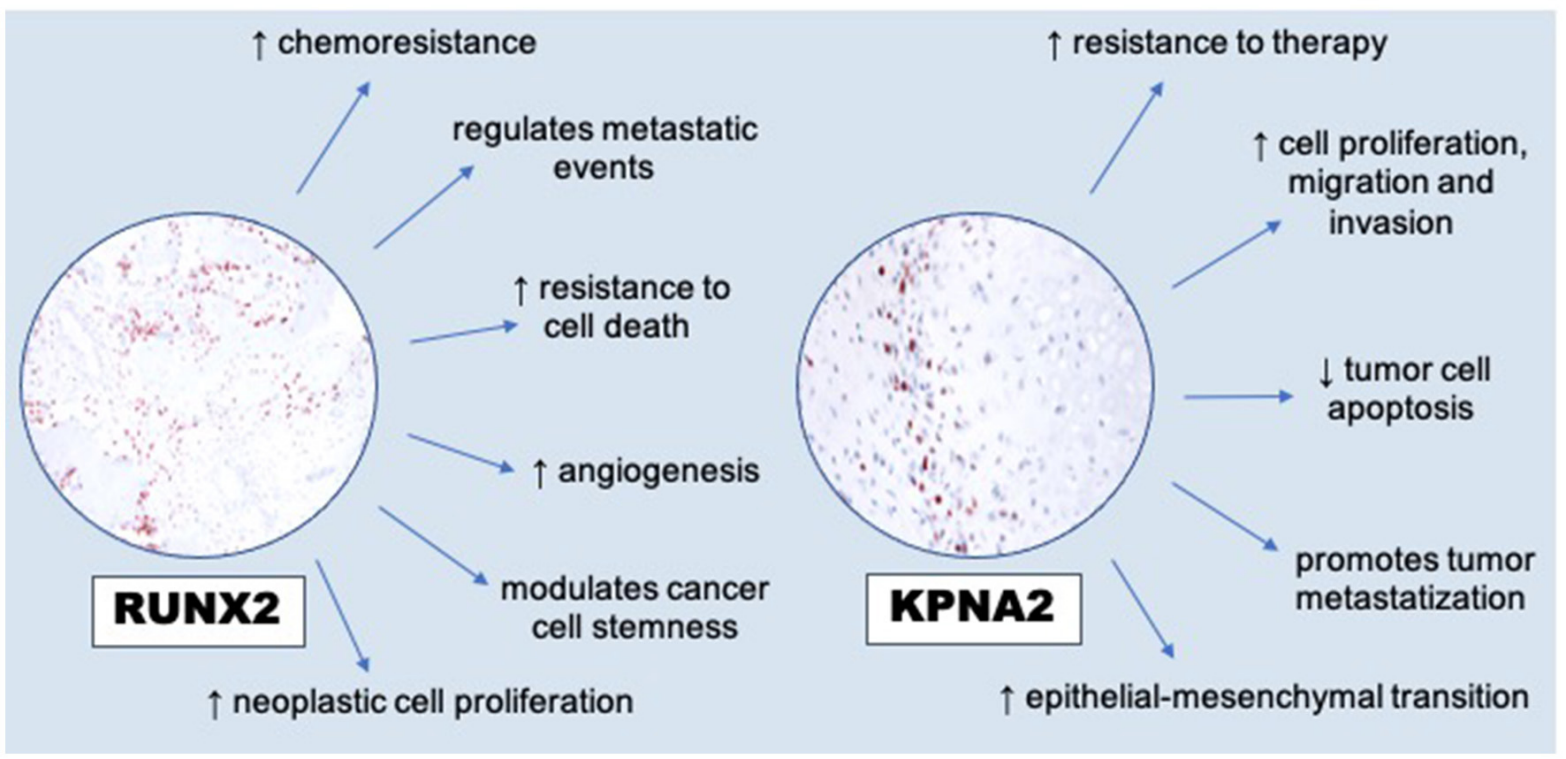
RUNX2′s modulations to hallmarks of cancer [modified from Lin (10)] and dysregulation of KPNA2 (overexpression) in promoting cancer cells [modified from Han and Wang (17)].

The interesting results obtained regarding the increased cellular expression of RUNX2 in neoplastic cells compared to the more dispersed and scattered expression of karyopherin alpha2 led us to consider these aspects also based on the different cellular immunoreactivity. In fact, the data relating to the more intense RUNX2 immunoreactivity of osteoblasts in the areas close to the osteoid deposits as opposed to that of karyopherin alpha 2 strongly encourage a more sophisticated continuation of the investigative work and insights into the complex nature and heterogeneous behavior of all the different forms of osteosarcoma. Even if the histological diagnosis and, to some extent, clinical and radiological aspects remain the diagnostic principles for osteosarcomas, immunohistochemistry can sometimes prove of help in defining the diagnosis and markers as karyopherin alpha-2 and RUNX2 seem to represent additional diagnostic tools to improve the specificity and sensibility in diagnosing osteosarcoma, even when extraskeletal (7, 14, 15). Karyopherin alpha-2, turned out to be a very interesting marker for diagnostic purposes, as it was always significantly expressed in all the extraskeletal osteosarcomas investigated. These data are in agreement with those already reported in the literature by Jiang et al. (7), who described a higher KPNA2 positivity in human osteosarcoma cases compared to other bone tumors such as chondrosarcoma, which is often considered in among the differential diagnoses (8). In particular, our preliminary investigation shows that also in extraskeletal osteosarcomas the expression of RUNX2 and karyopherin alpha-2 can represent specific diagnostic support for the definitive diagnosis of osteosarcoma, which is frequently made difficult by a set of factors including the frequent degree of undifferentiation, the heterogeneity of cellular atypia, and the difficulty in finding morphological elements characterizing this variable type of tumor, even in sites of primary onset, such as the extra-skeletal sites (5). The same reasoning must be carried out with the results obtained for RUNX2, a transcription factor of the RUNX family also responsible for various cellular processes, including cellular proliferation and differentiation, modulation of osteoblasts and chondrocyte differentiation with fundamental phases in skeletal development. As also reported by Barger et al. (13) in an interesting work on osteosarcomas published in Veterinary Pathology, in the absence of osteoid it is very difficult to distinguish some forms of osteosarcoma from other bone tumors. This also applies to extraskeletal osteosarcomas which frequently originate primarily from soft tissues, especially those of the mammary gland and manifest as poorly differentiated sarcomats, which are difficult to classify due to the absence of characterizing morphological features such as osteoid. Recent studies, such as that of Lin (10), also report on the critical role of RUNX2 in the dynamics of progression in different tumor types (16). It has been reported that RUNX2 may participate in the modulation of several key processes in cancer progression, including transdifferentiation and cancer stem cell potential, angiogenesis, proliferation and metastasis mechanisms, and potential drug resistance (Figure 3). These experimental observations suggest a multifunctional role of RUNX2 in the biological dynamics of cancer, although a potential protective role of RUNX2 in some cancers has been hypothesized by Wang et al. (4). It is important to underline that many of these variables associated with the potential biological activities of RUNX2 remain unknown and much more will need to be explored with future and increasingly specific further studies. We have certainly already set up new and upcoming investigative studies of this kind on a series of major EOSs and in association with other markers that may be involved together with RUNX2 and karyoperin alpha 2 in the etiopathogenetic mechanisms of both EOSs and SOSs.

## Data availability statement

The raw data supporting the conclusions of this article will be made available by the authors, without undue reservation.

## Ethics statement

Ethical approval was not required for the study involving animals in accordance with the local legislation and institutional requirements because, we apply investigations only in fixed-embedded tissues from our case bank, previously used for diagnostic investigations at University of Perugia.

## Author contributions

LL: Conceptualization, Funding acquisition, Supervision, Writing – original draft. EM: Conceptualization, Writing – original draft. AB: Conceptualization, Funding acquisition, Writing – review & editing. IP: Conceptualization, Investigation, Writing – original draft.
